# The Therapeutic Effects of Acupuncture and Electroacupuncture on Cancer-related Symptoms and Side-Effects

**DOI:** 10.7150/jca.55803

**Published:** 2021-10-11

**Authors:** Qiu-Qin Han, Yi Fu, Jia-Mei Le, Yu-Jie Ma, Xin-Dong Wei, Hou-Lin Ji, Haochen Jiang, Yueqiu Gao, Hailong Wu

**Affiliations:** 1 Shanghai University of Medicine & Health Sciences Affiliated Zhoupu Hospital, Shanghai 201318, China.; 2 Shanghai Key Laboratory of Molecular Imaging, Shanghai University of Medicine & Health Sciences, Shanghai 201318, China.; 3 Collaborative Innovation Center for Biomedicine, Shanghai University of Medicine & Health Sciences, Shanghai 201318, China.; 4 Institute of Clinical Immunology, Department of Liver Diseases, Central Laboratory, Shuguang Hospital Affiliated to Shanghai University of Traditional Chinese Medicine, Shanghai 200021, China.; 5 Laboratory of Cellular Immunity, Shuguang Hospital Affiliated to Shanghai University of Traditional Chinese Medicine, Shanghai 200021, China.

**Keywords:** acupuncture, electroacupuncture, cancer, surgery, chemotherapy, pain

## Abstract

In addition to cancer-related death, malignant progression also leads to a series of symptoms and side-effects, which would detrimentally affect cancer patients' the quality of life, adversely influence their adherence to treatments, and, therefore, negatively affect their long-term survival. Acupuncture and electroacupuncture (EA), as two classic treatment methods in traditional Chinese medicine, have been widely employed to cure various diseases. Recently, the clinical application of acupuncture and EA in cancer patients has received great attention. In this review, we summarized the clinical application of acupuncture and EA in alleviating the cancer symptoms, reducing the cancer treatment-related side-effects, and relieving the cancer pain. The symptoms and side-effects discussed in this review include fatigue, insomnia, chemotherapy-associated dyspepsia syndrome (CADS), pain, xerostomia, and anxiety and depression. The underlying mechanisms of the therapeutic effects of acupuncture and EA might be related to the regulation of the mitochondrial function, coordination of the activity of the nervous system, adjustment of the production of neurotransmitters, and alleviation of the immune responses. In conclusion, acupuncture and EA have been proved to be beneficial for cancer patients. More research, however, is required to clarify the potential mechanisms behind acupuncture and EA for widespread adoption in clinical application.

## Introduction

Carcinoma is the leading cause of global morbidity and mortality, so cancer prevention and treatment have become the key research topic in the medical field. In addition to death, the progression of cancer is also accompanied by a series of symptoms and side-effects that is caused by not only cancer itself but also cancer-related therapies, such as surgery, chemotherapy, radiotherapy, etc. These symptoms and side-effects will severely impact the life quality and treatment adherence of cancer patients, and, therefore, adversely affect their long-term survival. Recently, complementary and alternative medicine (CAM), including acupuncture, deep breathing exercises, massage therapy, meditation, naturopathy, and yoga, has been increasingly adopted by cancer patients for seeking relief from cancer-associated symptoms 1. Among them, acupuncture, a well-practiced therapeutic approach in traditional Chinese medicine, has been implicated as an effective approach in improving cancer-associated symptoms 1.

Acupuncture has been defined as the insertion of fine needles into specific acupuncture points (acupoints) in the human body. In Eastern Asia, acupuncture has been widely employed to treat various diseases for over 2,500 years. Notably, the benefits of acupuncture in treating various diseases have been gradually recognized by Western society. For example, the World Health Organization (WHO) and the National Institutes of Health (NIH) released a report on acupuncture in 2003, suggesting more than 100 types of diseases and conditions could be treated by acupuncture 2; the NIH issued a Consensus Statement on Acupuncture to support the use of acupuncture for adult postoperative and chemotherapy-associated nausea and vomiting, postoperative dental pain, addiction, stroke rehabilitation, headache, menstrual cramps, fibromyalgia, myofascial pain, osteoarthritis, low back pain, carpal tunnel syndrome, and asthma 3; the FDA had approved acupuncture needles for use by licensed practitioners 4; the number of total licensed acupuncturists has increased 257% from 1998 to 2018 in the United States 5. Recently, a study on integrative oncology (IO) service in North America, Europe, and Australia showed that acupuncture is one of the most frequent IO services in the United States and the European Union 6.

Electroacupuncture (EA) is developed around the mid-1900s and is a modified approach of acupuncture that applying weak electronic currents through the needles after conventional acupuncture procedure. Although it is still controversial, EA has been shown to achieve similar or even better effects compared to acupuncture. Moreover, the efficacy of acupuncture mainly depends on the manipulation technique of the acupuncturist, but EA can be practiced more reproducibly and is more suitable for both basic and clinical research. In this review, we introduced the effects of acupuncture and EA on cancer-related symptoms and side-effects, the possible mechanisms behind acupuncture, and the hindrance against the wide application of acupuncture and EA in cancer-related treatment.

## Effects of acupuncture and EA on the symptoms and side-effects related to cancer and cancer treatment

Cancer and cancer treatments are frequently associated with a lot of symptoms and side-effects including fatigue, insomnia, chemotherapy-associated dyspepsia syndrome (CADS), radiation-induced xerostomia (RIX), pain, vomit and nausea, cognitive impairment, distress, anxiety, depression, etc. Because some of those symptoms and side-effects are tightly related, we mainly discussed the therapeutic potential of acupuncture and EA in some of those symptoms and side-effects in this review.

### Fatigue

Cancer-related fatigue (CRF) has been recognized as one of the most common cancer-related side-effects 7 and usually cannot be alleviated by adequate sleep or rest 8. The onset of CRF commonly occurs before the cancer treatments and it frequently gets worse during the therapies 9, 10. Although cancer patients usually will be relieved from CRF after treatment completion, near 25% to 30% of cured patients still suffer persistent fatigue for up to 10 years 11, 12. More importantly, CRF has been recognized as a risk factor for short survival in cancer patients 13, 14.

The effective treatment options for CRF are limited, consisting of non-pharmacologic interventions like physical activity and psychosocial and mind-body interventions. However, a recent clinical trial, including 302 breast cancer outpatients, showed that acupuncture could significantly relieve CRF 15. Meanwhile, EA has been shown to significantly improve fatigue in breast cancer patients treated with aromatase inhibitors 16, 17. In addition, acupuncture has been shown to improve fatigue in lung cancer patients 18 and head and neck cancer (HNC) patients, who were subjected to chemoradiation therapy 19. Furthermore, a meta-analysis study on ten randomized controlled trials confirmed acupuncture as an effective means for CRF 20. Therefore, acupuncture and EA could serve as an effective and feasible approach to relieve CRF.

### Insomnia

Insomnia, also called sleeplessness, is recognized as the most prevalent sleep problem worldwide. The reported prevalence of insomnia in cancer patients is up to 50%, which is three times higher than that in the general population 21. As cancer-related insomnia (CRI) is frequently viewed as a normal and transient response to cancer itself or cancer treatment, CRI is commonly neglected by both clinicians and cancer patients, leading to having a chronic insomnia symptom in a substantial proportion of cancer patients 22-24. CRI could be treated by both pharmacologic and non-pharmacologic approaches. Hypnotic agents, including Benzodiazepines (BZDs) and non-BZDs agents, and antidepressants, such as amitriptyline, doxepin, mirtazapine, and trazodone, are commonly used in the pharmacologic treatment of CRI 25. Although medication could improve sleep outcomes in the short-term, the significant side-effects and the concerns of drug-drug interactions limit their use. Meanwhile, as the non-pharmacologic approaches, acupuncture and EA have been shown to be effective for treating primary insomnia 26, insomnia after stroke 27, menopause insomnia 28, and chronic pain-related insomnia 29, but whether they are effective to treat CRI remains controversial. A previous study recruited 6 randomized controlled trials only showed a low level of evidence supporting the therapeutic advantage of acupuncture in the treatment of CRI 30. On the contrary, other studies showed that acupuncture could produce meaningful and durable improvements in CRI in cancer survivors 31 and patients 32. However, all of these studies suffer the weakness of a small sample size, so more adequately powered clinical trials are needed to clarify the therapeutic efficacy of acupuncture and EA in treating CRI.

### Chemotherapy-Associated Dyspepsia Syndrome (CADS)

Chemotherapy is routinely employed in combination with radiotherapy, surgery, hormone therapy, or immunotherapy for treating many types of cancers. Despite the effectiveness in reducing tumor burden, chemotherapy also unavoidably raises many irreparable side-effects, such as chemotherapy-associated dyspepsia syndrome (CADS). CADS is defined as a collection of gastrointestinal symptoms after receiving chemotherapy, including early satiety, anorexia, diarrhea, nausea, and vomiting 33. It has been reported that up to 20% of cancer patients had to postpone or even terminate potentially curative chemotherapy due to CADS 34. Although prophylactic administration of antiemetics can reduce nausea and vomiting in 70-90% of cancer patients 35, these antiemetics have limited effects on chemotherapy-induced early satiety, anorexia, and diarrhea.

Acupuncture has been commonly employed in China to treat gastrointestinal symptoms for thousands of years and the possible mechanisms behind might be through the altering acid secretion, GI motility, and visceral pain 36. A previous study has demonstrated that chronic EA at ST36 can improve cisplatin-induced dyspepsia symptoms and gastric dysmotility in rats probably by enhancing vagal efferent activity and reducing satiety hormones 37. In addition, EA at CV12 has shown to be effective to improve cisplatin treatment-induced anorexia in rats 9. More importantly, several randomized multicenter crossover studies have shown that acupuncture could serve as a supportive antiemetic approach to reduce the medical use of antiemetics in pediatric cancer patients, who are receiving highly emetogenic chemotherapy 38, 39. Intriguingly, a single-blind, randomized, and controlled clinical trial with a total of 142 liver cancer patients enrolled showed that transcutaneous electrical acupoint stimulation (TEAS) significantly improved cisplatin-induced anorexia compared to placebo controls 40. Therefore, evidence from both experimental studies and clinical trials supports acupuncture and EA as a feasible and effective approach to alleviate CADS.

### Pain Related to Cancer and Cancer Treatments

Pain is the most common symptom of cancer and is reported in 90% of cancer patients at various progression stages 41. Moreover, moderate to severe pain has been claimed in 40% of patients carrying early or intermediate stage cancer and 80% of cancer patients at advanced stages 42, 43. More importantly, near 70% of cancer pain is undertreated 43.

Acupuncture and EA have been widely employed to relieve non-malignant acute and chronic pain 44. Recently, acupuncture and EA-induced analgesic effect has been reported in both experimental cancer models and cancer patients. EA at GB30 and ST36 have been reproducibly shown to improve cancer-induced hyperalgesia in the rat bone cancer model by multiple groups 45-47. EA at Baihui, Quchi, Neiguan, Xuehai, Zusanli, and Sanyinjiao have been shown to alleviate cancer pain in patients with advanced hepatocellular carcinoma 48. A case report suggested that acupuncture may greatly improve the neuropathic pain induced by bone metastasis in patients with advanced cancer^49^. A recent case report study demonstrated that acupuncture is an effective and safe therapeutic option for reducing cancer pain with minimal side-effects and lowering the need for narcotic analgesics 50. A randomized controlled clinical trial demonstrated that EA at Jiaji (Ex-B2) from T8 to T12 bilaterally could significantly relieve pancreatic cancer pain 51. In addition to the analgesic effect on cancer pain, acupuncture and EA were also shown to improve pain caused by cancer treatments, such as surgery and chemotherapy. EA at Neimadian (Extra) and Neiguan (PC 6) have been shown to improve post-operation pain in esophageal cancer patients, who underwent thoracic surgery 52. A previous study has shown that EA significantly inhibits allodynia and hyperalgesia in an established rat model of paclitaxel-induced peripheral neuropathy 53. EA has been reported to improve thalidomide- or bortezomib-induced peripheral neuropathy in patients with multiple myeloma 54. Moreover, a very recent randomized controlled pilot trial demonstrated that acupuncture could significantly improve taxane-induced peripheral neuropathy in breast cancer patients 55. Therefore, multiple lines of evidence have indicated that acupuncture and EA could serve as complementary therapy to relieve pain that is associated with cancer and cancer treatments.

### Xerostomia

Salivary glands are significantly sensitive to radiation therapy and will be irreversibly damaged at doses higher than 50 Gy 56. It has been reported that more than half of the patients, who received radiation therapy involving major salivary glands, experienced hyposalivation, a symptom also termed radiation-induced xerostomia (RIX), by the end of treatment 56. RIX is a common and often debilitating adverse effect of radiation therapy among patients with head and neck cancer. A randomized clinical trial found that acupuncture resulted in significantly fewer and less severe RIX symptoms 1 year after the treatment compared to standard care control 57.

### Anxiety and Depression

Anxiety and depression are common complications of cancer, influencing cancer patients' quality of life, their adherence to treatment, and their survival 58, 59. The prevalence rates of anxiety and depression in cancer patients were 19% and 12.9% respectively 60. The results regarding whether acupuncture could improve cancer-related anxiety and depression are controversial. A clinical trial with 47 enrolled breast cancer patients (23 for real-acupuncture vs. 24 for sham-acupuncture) revealed that acupuncture has no significant effect to improve anxiety and depression associated with the treatment of aromatase inhibitors (AIs) 61; whereas another clinical trial showed that EA could significantly improve anxiety and depression associated with AIs treatment in breast cancer patients 16. Meanwhile, a clinical trial including 302 breast cancer patients showed that acupuncture could improve cancer-related anxiety and depression 15. It is worth noting that, in all three clinical trials above, anxiety and depression are the secondary measurement outcomes. One study, using depression as the primary outcome, illustrated that acupuncture can effectively reduce malignancy-related depression 32. In summary, large clinical trials assessing anxiety and depression as the primary outcome need to be conducted to evaluate the role of acupuncture in cancer-related anxiety and depression.

## Mechanisms Related to the Therapeutic Effects of Acupuncture and EA

Although many efforts have been made to understand the effects of acupuncture and EA in the treatment of cancer-related symptoms and side-effects, the mechanisms by which acupuncture and EA achieve therapeutic benefits remain largely unknown. The main focus has been on investigating the mechanisms of the analgesic effects of acupuncture and EA. In the early 1970s, Han's group, for the first time, strongly suggested the involvement of central chemical mediators in the analgesic effect by acupuncture. They transferred the cerebrospinal fluid of acupunctured donor rabbits into recipient rabbits and achieved analgesic effects in recipients 62. From then on, a series of studies have demonstrated that the analgesic effects may be attributed to the neurotransmitters induced by acupuncture and EA. For example, naloxone, a specific opioid receptor antagonist, had been shown to partially reverse the analgesic effect of acupuncture on electrical stimulation-induced tooth pulp pain and chronic pain in humans and monkey subjects 63, 64; poor EA-induced analgesia had been observed in CXBK mice that are deficient with opioid receptors 65; protection of endogenous opioid peptides by using peptidase inhibitors could potentiate acupuncture analgesia 66, 67. In addition, some clinical trials have demonstrated that acupuncture and EA could significantly decrease the expression of substance P to relieve pain in patients with fibromyalgia 68, acute herpes zoster 69, knee osteoarthritis 70, and so on. Moreover, EA has been shown to relieve the pain of knee osteoarthritis by enhancing the response of serotonin via the upregulation of serotonin receptor 2A/C 71.

The studies on mechanisms of acupuncture and EA for treating other cancer-related symptoms and side-effects are limited. A previous study has shown that EA can improve chronic fatigue by reducing mitochondrial oxidative stress and increasing ATP synthesis 72. Acupuncture and EA have been shown to improve insomnia by reducing the sympathetic nervous activity 73, suppressing the activation of the hypothalamic-pituitary-adrenal (HPA) axis 74, 75, increasing the levels of Gamma-aminobutyric acid (GABA) and GABA(A) receptor 76, and elevating the generation and secretion of melatonin 77. EA at CV12 has been shown to improve CADS via an increase in the secretion of ghrelin and cholecystokinin (CCK) and a decrease in the secretion of 5-hydroxytryptamine (5-HT) into the serum 9, 78. In addition, chronic EA at ST36 has been shown to improve cisplatin-induced dyspepsia via modulating the production of the vagal and gastrointestinal hormones including fasting ghrelin, glucagon-like peptide-1, and peptide YY 37.

## Summary

This review summarized the application of acupuncture and EA in cancer patients for improving cancer-related symptoms and side-effects, such as fatigue, insomnia, chemotherapy-associated dyspepsia syndrome (CADS), radiation-induced xerostomia (RIX), anxiety and depression, and pain (**Table 1**). We also simply introduced the potential mechanisms involved in the therapeutic effects of acupuncture and EA (**Table 2**). Given that the actual mechanisms of acupuncture and EA remain largely unknown, more basic and clinical studies are needed to endorse the broad clinical application of acupuncture and EA in the treatment of cancer-related symptoms and side-effects.

## Figures and Tables

**Table 1:** The therapeutic effects of acupuncture and electroacupuncture in treatment of cancer-related symptoms and side-effects

Therapeutic Effects	AC or EA	Study subjects	Acupoints	Ref
Anti-fatigue	AC	Cancer-related fatigue in breast cancer	ST36, SP6, LI4, GB34, SP9	15
	EA	Fatigue in breast cancer patients with aromatase inhibitor-related arthralgia	N/A	16,17
	AC and EA	Fatigue in head and neck cancer patients with chemoradiation therapy	ST36, SP6, LI2, LI11, GV20, Shenmen/ear, Sanjiao/ear, ST7, ST6, ST5, CV23, GB20, EX-HN3	19
	AC	cancer-related fatigue in lung cancer patients	LI4, REN6, ST36, KI3, SP6	18
Anti-insomnia	AC	Insomnia in cancer survivors	HT7, SP6, GV20, GV24, Shenmen (Auricular), Sympathetic (Auricular)	31
	AC	Insomnia in cancer patients	ST40, SP9, SP10, SP6, EX-HN3, DU20, EX-HN1, PC6, TF4	32
Anti- chemotherapy-associated dyspepsia syndrome (CADS)	EA	A rodent model of dyspepsia induced by cisplatin	ST36	37
	EA	A rat model of cisplatin-induced anorexia	CV12	9
	AC	Chemotherapy-induced nausea and vomiting in pediatric cancer patients	Point combinations depended on the acupuncturist's decision. Most commonly used points were PC6, ST36, CV12, LI4.	38,39
	EA	Liver cancer patients who receive chemotherapy via intravenous infusion or transcatheter arterial chemoembolization (TACE)	PC6, ST36, CV12	40
Analgesia	EA	A rat bone-cancer pain model	ST36, GB30	45-47
	EA	Cancer pain in patients with advanced hepatocellular carcinoma	GV20, LI11, PC6, ST36, SP6	48
	AC	Neuropathic pain induced by cancer bone metastasis	GB29, GB30, GB34, GB40, BL40, ST36	49
	EA	Pancreatic cancer pain	Ex-B2 points from T8 to T12 bilaterally	51
	EA	Postoperative pain in esophageal cancer patients	EX28, PC6	52
	EA	Paclitaxel-induced peripheral neuropathy in a rat model	GB30	53
	EA	Thalidomide/bortezomib-induced peripheral neuropathy in cancer patients with multiple myeloma	LI4, SI3, Baxie 2, Baxie 3, LV3, SP6, GB42, ST36, Bafeng 2, Bafeng 3, DU20, CV4, CV6	54
	EA	Chemotherapy induced peripheral neuropathy in breast cancer survivors	SP9, ST36, SP6, K3, LR3, LI11, TW5, Baxie, Yintang	55
Anti-xerostomia	AC	Xerostomia in patients with head and neck cancer	Ren 24, LU 7, K6, Shenmen, Point Zero, SG 2-prime, Larynx	57
Anti-anxiety and depression	AC	depression in patients with malignant tumor	ST 40, SP 9, SP 10, SP 6, EX-HN3, DU 20, EX-HN1, PC 6, Shenmen	32

AC: Acupuncture; EA: Electro-acupuncture.

**Table 2:** Mechanisms related to the therapeutic effects of acupuncture and electroacupuncture

Therapeutic Effects	AC or EA	Study subjects	Mechanisms	Ref
Anti-Fatigue	EA	Rats with chronic fatigue syndrome	Reducing mitochondrial oxidative stress and increasing ATP synthesis	72
Anti-insomnia	AC and EA	Insomnia in stroke patients	Reducing the sympathetic nervous activity	73
	AC	Insomnia in chronic stress rats or in maternal separation rats	Suppressing the activation of hypothalamic-pituitary-adrenal (HPA) axis	74,75
	AC	Insomnia rats	Increasing the levels of Gamma-aminobutyric acid (GABA) and GABA(A) receptor	76
		Insomnia in anxious adult	Elevating the generation and secretion of melatonin	77
Anti- chemotherapy-associated dyspepsia syndrome (CADS)	EA	A rat model of cisplatin-induced anorexia	Increasing the secretion of ghrelin and cholecystokinin (CCK) and decreasing the secretion of 5-hydroxytryptamine (5-HT) into the serum	9,78
	EA	A rodent model of dyspepsia induced by cisplatin	Modulating the production of the vagal and gastrointestinal hormones	37
Analgesia	AC and EA	Electrical stimulation-induced tooth pulp pain in humans and monkey	Inducing endogenous opioid peptides and enhancing opioid-opioid receptor signaling	63,64
	EA	CXBK mice		65
	AC and EA	Pain induced by radiant heat exposure in rabbits		66,67
	AC	Pain in patients with fibromyalgia	Decreasing the expression of substance P	68
	AC	Pain in patients with acute herpes zoster		69
	EA	Pain in patients with knee osteoarthritis		70
	EA	Pain in an Osteoarthritis Rat Model	Enhancing response of serotonin via upregulation of serotonin receptor 2A/C	71

AC: Acupuncture; EA: Electroacupuncture.
